# Centrifugal Microfluidics Traps for Parallel Isolation and Imaging of Single Cells

**DOI:** 10.3390/mi11020149

**Published:** 2020-01-29

**Authors:** Adam Snider, Ileana Pirozzi, Anubhav Tripathi

**Affiliations:** Center for Biomedical Engineering, School of Engineering, Brown University, Providence, RI 02912, USA; adam_snider@brown.edu (A.S.); ileana_pirozzi@brown.edu (I.P.)

**Keywords:** microfluidics, single cell, cell isolation, centrifugal, cell imaging

## Abstract

Analysis at the single cell level has becoming an increasingly important procedure to diagnose cancer tissue biopsies. These tissue samples are often heterogeneous and consist of 1000–15,000 cells. We study the use of centrifugal microfluidics to isolate single cells into micro chambers. We describe the optimization of our microfluidics flow device, characterize its performance using both polystyrene beads as a cell analogue and MCF-7 breast cancer cells, and discuss potential applications for the device. Our results show rapid isolation of ~2000 single cell aliquots in ~20 min. We were able to occupy 65% of available chambers with singly occupied cancer cells, and observed capture efficiencies as high as 80% using input samples ranging from 2000 to 15,000 cells in 20 min. We believe our device is a valuable research tool that addresses the unmet need for massively parallel single cell level analysis of cell populations.

## 1. Introduction

The understanding of a tissue as a heterogeneous group of cells has driven the growing research and clinical interest in studying populations of cells at single cell resolution. Imaging of a large number of single cells is relevant to developmental biology, histology, neuroscience, immunology, physiology, functional genomics, stem cell research, and cancer research [1]. This wide array of applications share similar criteria, the system must be maintain cell viability and the system must analyze a large population of cells at single cell resolution so rare cell types do not disappear into the noise of the rest of the sample [1]. Other desirable criteria include the ability to perform the cell separation and imaging on the same device, avoiding the need to manually pipette the cells, and also making the device simple to fabricate, and inexpensive to make and operate, so that it is accessible to more laboratories and facilities [1].

Therefore, a variety of strategies have emerged for isolating and imaging a large number of individual cells. Solid capture devices rely on the attachment of the target cells to a surface, thereby making the cells unrecoverable [2]. Furthermore, these systems are marker-dependent, capturing only cells expressing specific surface markers rather than all cells in a population [2]. Solid-phase capture devices do, however, allow for a series of reagents to be applied to the cells via a flow cell [2]. Groove-chips have been used to achieve cell alignment. They are easy to fabricate, are compatible with fluorescent and bright field microscopy, and are compatible with a wide range of cell lines, but do not achieve single cell resolution [3]. The Mother Machine has been used by a host of labs to study rapidly dividing microbial cells by loading them into single-opening or double-opening channels and has been implemented with a high-degree of automation [4,5]. However, most of this work is specific to growth studies performed on bacterial samples and would not be applicable to human cell lines due to difficulty loading the cells. Another category of devices use dielectrophoresis to create an electroactive microwell array (EMA), however these types of arrays require pre-alignment focusing of the cells before loading onto the array [6]. The integration of a focusing device, such as a flow-based acoustophoresis device, on top of the EMA requires very complex device fabrication and alternate tuning and operation for different cell lines with alternate acoustic or dielectrophoretic properties [6]. Fluorescent-activated cell sorting (FACS) [7] is commonly used to isolate specific cell types from a large input of cells, often exceeding 10,000 cells.

Song et al. described an interesting microfluidic device allowing for monitoring a single cell under static or dynamic media concentration profiles, but the device is not multiplexed nor does it confine the cell to a fixed well on the microfluidic chip, thereby requiring a microscope capable of cell tracking [8]. Other microfluidic devices are capable of multiplexed single cell imaging and analysis, but require an optical trap for loading the cells into the wells, and are thereby very labor intensive [9]. Laser capture microdissection (LCS) uses microscopy that directly captures single cells from cell suspensions or solid tissue samples [10].

Here, we designed a centrifugal flow device with microfluidic cell traps to enable isolation of single cells for subsequent analyses or potential micropipette-based recovery. Our device offers several advantages compared to the other microfluidic devices mentioned earlier. The flow device allows parallel monitoring of multiple, separate, individual cells, while maintaining high trap occupancy efficiency. The device exploits centrifugal fluid transportation to regulate pumping pressure and prescribe body forces on cells immersed in a fluid, allowing for efficient control of the cell movement in a radial direction. In the next section we describe materials and methods followed by the results and discussion sections.

## 2. Materials and Methods

**Figure 1:**
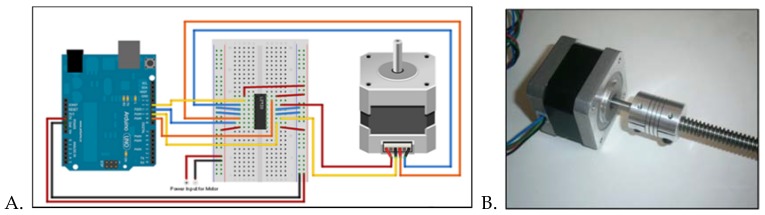

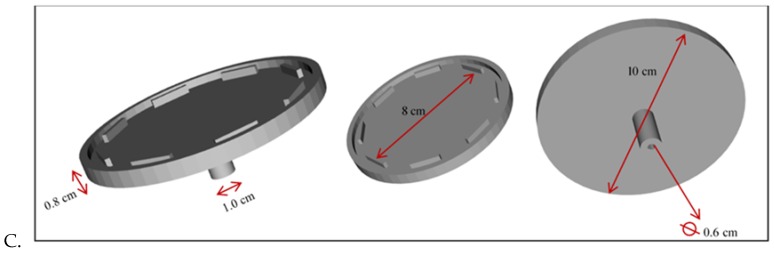
(**A**) Connection diagram of stepper motor (**right**), Arduino UNO (**left**), motor driver and breadboard (center). (**B**) Assembly of stepper motor shaft with lead screw via a coupler. (**C)** Angled views of the mounting plate. A hole (diameter 6 mm) was extruded through the extension connected to the mounting platform in order to fit the motor’s shaft. A set of blockers (1 mm thick, 1 cm wide) were arranged octagonally around an 8 cm diameter and were extruded 5 mm from the base of the platform. The polydimethylsiloxane (PDMS) disc fits inside the octagonal blockers.

**Device Assembly:** The microfluidic platform was designed in SolidWorks (Dassault Systèmes, Vélizy-Villacoublay, France). From the calculations made in the *Modeling* section, the useful range of speeds is 0–200 rpm. Due to the lack of commercial availability of turntables in this speed range, it was decided that the device would be assembled using a stepper motor, which can supply the required angular velocity. The bipolar stepper motor (Stepperonline, Nanjing, China) is powered and controlled through an Arduino UNO (Digi-key Electronics, Minneapolis, MN, USA), as shown in Figure 1A. The stepper motor was connected to the Arduino through a motor IC driver, as shown in the diagram below (L293D DIP/SOP Push-Pull Four-Channel Stepper Motor Driver IC Chip, CNUS, Digi-key Electronics, Minneapolis, MN, USA). The speed of the motor was programmed in Arduino UNO (Digi-key Electronics, Minneapolis, MN, USA).

**Figure 2:**
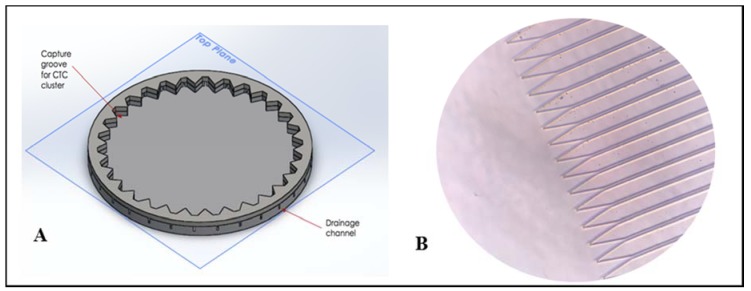
(**A**) CAD drawing of microfluidic centrifugal device (MCD) for circulating tumor cells (CTC) cluster capture and analysis. Note: Figure not to scale. The real device has a radius of 36.5 mm and a total of 1000 wells along the circumference. (**B**) Micrograph (20X) of individual traps and corresponding channels. Scale: 30 µm is the distance from one capture well to another.

The motor shaft (5 mm diameter) was then connected to a twisted screw via a coupler (Figure 1B) in order to be connected to a custom mounting plate designed in SolidWorks 2016 (Figure 1C). The mounting plate was designed to hold the polydimethylsiloxane (PDMS) microfluidic platform and connect the stepper motor’s lead screw to the microfluidic centrifugal device (MCD), fabricated using method described below. Figure 2 shows a Solidworks drawing of the microfluidic platform, alongside a micrograph, using Leica DM 5500B (Leica Microsystems, Buffalo Grove, IL, USA), of a section of the device. 

The apparatus was connected to a power supply and operated at 9V and 1A. The Arduino was connected to a desktop computer in order to easily control the rotational speed. Note: the PDMS platform is covered by a glass top piece.

**Microfluidic Device Fabrication:** A Solidworks-designed master mold was obtained by SU-8 photolithography (Flowjem Inc., Toronto, Canada) and it was used to create PDMS MCDs. Sylgard 184 elastomer base was vigorously mixed at a 1:10 mass ratio, for a total mass of 55 g. The mixture was degassed in a vacuum chamber for 1 h, poured into the mold and then incubated at 70 °C for one hour. A 2 mm-thick glass slide and the PDMS were cleaned with methanol and diH_2_O, dried with nitrogen, and bonded by applying gentle pressure.

**Device Operation:** The MCD was prepared by running 1 mL of suspension buffer (no cells) through the central chamber for 5 min at 60 rpm. Then monodisperse cell suspensions were prepared containing 2000–50,000 cells in volumes ranging from 500 μL to 1500 μL. The suspension was loaded dropwise onto the central chamber using a pipette. The cell-suspension drops were distributed evenly along the circumference of the disk at a loading radius of 2 cm (see the Mathematical Modeling section). After loading the cell suspension, the MCD was topped with a custom glass piece, which was gently pressed on the PDMS platform. The device was then powered at 9 V, and the stepper motor was operated at frequencies varying from 50–200 rpm, with stepwise and/or gradual frequency increments. The running time was optimized for maximal trapping frequency.

**MCF-7 Cell Culture:** MCF-7 (ATCC, Manassas, VA, USA) cells were cultured in Eagle’s Minimum Essential Medium (ATCC, Manassas, VA, USA) (EMEM supplemented with 10% (v/v) Fetal Bovine Serum (FBS) (Corning, Corning, NY, USA) and 1% v/v penicillin streptomycin) at 37 °C under 5% CO_2_. The cells were grown to 90% confluence before passaging. To generate MCF-7 clusters, we trypsinized MCF-7 cells growing in monolayer at 80% confluence for 1 min to gently generate floating clusters. The size of the clusters varied from 2 cells to ~15 cells per cluster.

**Cell Suspension Solution:** Buffers were prepared containing 0.3% Tween-20 (Sigma–Aldrich) and biotin-free BSA (10X) in phosphate-buffered saline (PBS, Thermo-Fisher Scientific, Waltham, MA, USA). This solution was used to suspend the single cells loaded onto the MCD. Time course microscopy was performed to ensure cell viability in this buffer. The buffer was designed to limit adhesion of cells to the walls of the device during cell transit from the middle of the device to the capture wells. The cells were trypsinized and the cells in the buffer were carefully resuspended by repeatedly pipetting until all cells appeared as single cells by microscopy.

**Obtaining Cell Counts:** A Leica DM 5500B microscope (Leica Microsystems, Buffalo Grove, IL, USA) using a 20x objective lens was used to take most of the microscopic observations. To quantify capture efficiency, counted using a hemocytometer and imaged the single cells captured on the MCD as well as those that remained in the loading chamber.

**Polystyrene Bead Testing:** Functionality of the device was first tested with 15 µm-diameter polystyrene microbeads (Polysciences Inc, Warrington, PA, USA). The beads were provided by the manufacturer at a density of 5 M/mL and were serially diluted to reach several concentrations ranging from 2000 to 15,000 beads/mL in PBS. Then, 1 mL of the suspension was loaded in the central chamber of the device.

## 3. Results and Discussion

**Figure 3:**
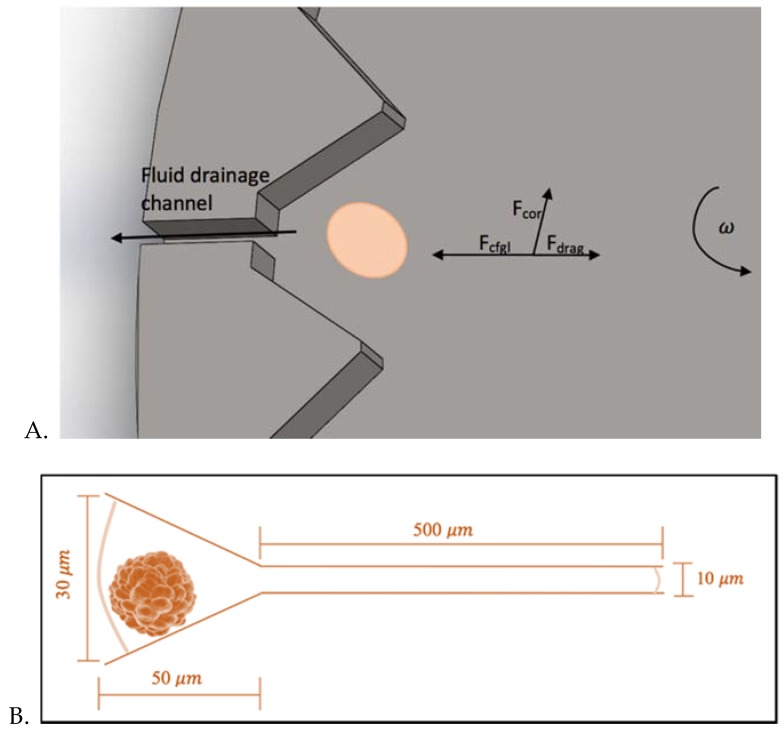
(**A**) Single functional unit of the MCD, featuring the cell trap and its connected microfluidic channel. (**B**) Single cell trap dimensions. Note: Figure not to scale.

**Microfluidic Centrifugal Device Design:** A primary design goal, was for the technology to be point-of-care, which is exemplified by the design of this simple, centrifugal, pumpless trapping device that allows parallel monitoring of multiple, separate, individual cells, while maintaining similar trap occupancy efficiency of other, more sophisticated devices. The portable, microfluidic centrifugal device exploits centrifugal fluid transportation to sophisticatedly control pumping pressure and impose body forces on cells immersed in a fluid, allowing for efficient control of the cell movement in a radial direction. The PDMS platform (10 cm in diameter) consists of a central loading chamber, where the cells are loaded in a monodisperse solution (Figure 2A) and an array of funnel-shaped traps aligned along the circumference of the chamber. The PDMS platform is then covered with a custom glass piece to enable the flow to be completely radial. As the centrifugal motion is actuated, the cells are guided towards 4000 individual traps, arranged along the circumference of the disk. Each trap (width −30 µm) was designed to only hold one cell. The device was designed specifically to capture MCF-7 cells, a breast cancer cell line of dimensions ranging from 18–20 µm [11] (Figure 3). Each trap is connected to its own pressure-activated drainage channel, of diameter 10 µm (Figure 2B). The channels are too narrow to allow for the passage of a cell, and they function as passive valves (Figure 6): the rotational motion exerts a certain pressure to the entrance of the trap and, and the fluid will flow through the channel as long as the centrifugal pressure is larger than the capillary pressure within the channel. The implication of this system is that the centrifugal pressure applied on the channels can be carefully prescribed by modifying the rotational frequency of the device; thus the cells can be exposed to physiological ranges of shear flows (as predicted computationally) and the fluids can be drained at will, by increasing the rotational frequency beyond a calculated burst frequency, which will enable the fluid to flow through the channels. The innovative use of these passive-valve drainage channels enables successful cell isolation, preventing cells from migrating back towards the center of the disk when the rotational motion is arrested. Once the cells are isolated and compartmentalized, they can undergo subsequent single cell analysis and on-chip assays: given that fluids can be drained, a sequence of fluids can be directed through the device such as lysing buffer antibody cocktails for multiplexed fluorescent microscopy.

It is important to note that balancing the burst pressure of the channels with the force generated by centrifugation is critical to the operation of this device. Needing to use lower angular velocities would increase the total operating time of the device. Making the cross-sectional area of the channels narrower would allow for shorter drainage channels but would make micropipetting more difficult. This work focuses on the isolation of single cell aliquots for imaging-based studies.

**Figure 4:**
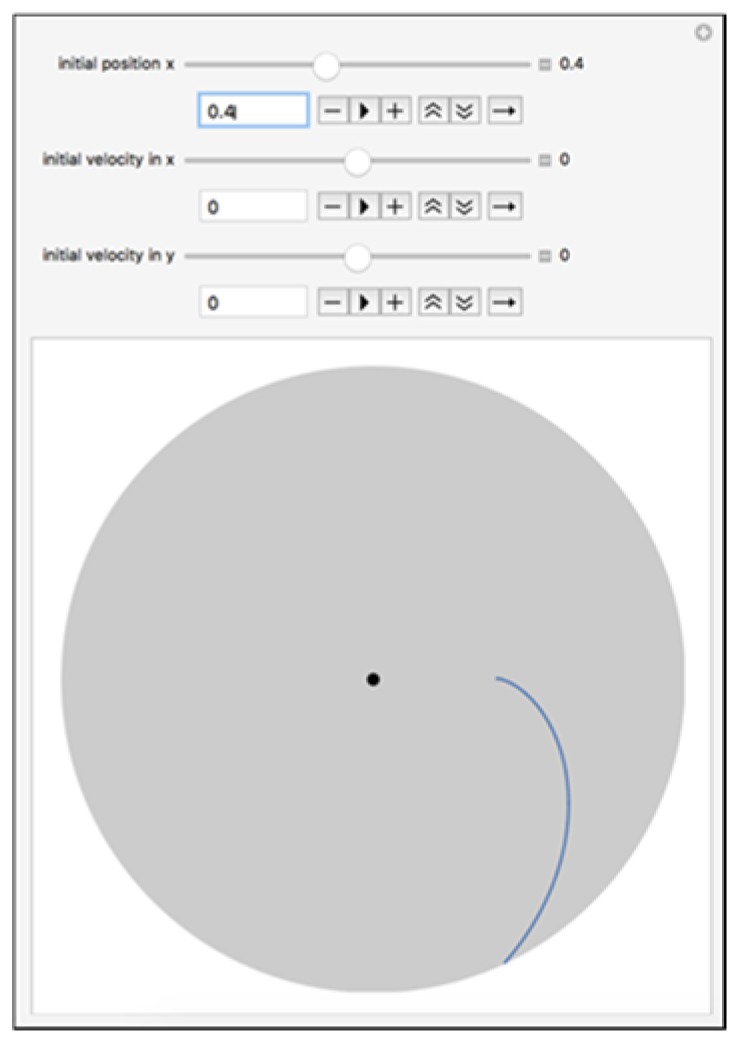
Trajectory of the cell with Ro = 2.0 cm. The model was created in Wolfram Mathematica in a computable document format.

**Theoretical Analysis of the Transport and Flow of Cells:** Three main forces are responsible for the movement and the trajectory of the cells from their initial position R_0_ to the traps on the circumference of the rotating platform: the centrifugal force (*f_c_*), the Coriolis force (*f_cor_*), and the drag force (*f_d_*). The combined action of these forces is responsible for the predicted curved trajectory of the cell, as shown in Figure 4**.** The Coriolis force is an effect whereby a mass moving in a rotating system experiences a force acting perpendicular to the direction of motion and to the axis of rotation [12]. This design takes advantage of this effect to ensure that cells are driven to the sides of the funnel-shaped traps (Figure 3A), leaving the drainage channels unclogged to allow for successful fluid drainage. From a force balance of the centrifugal, drag and Coriolis forces (and taking buoyancy into account), we can write the following expression for the sum of the forces on a single cell:

(1) $$\sum F_{r}=F_{cen}-F_{d}-F_{cor}=m\frac{du_{c}}{dt}$$

where m is the mass of a cell and $u_{c}$ is the cell velocity. Substituting in the expressions for the individual forces, we obtain the following:

(2) $$m\frac{du_{c}}{dt}=\frac{m\;r\omega^{2}\Delta \rho }{\rho_{c}}-6\pi \mu r_{c}u_{c}-2m\omega u_{c}$$

where $\rho_{c}$ is the density of the cell, $\Delta \rho =\rho_{c}-\rho_{f}$ is the difference between the cell and fluid density, μ is the viscosity of the fluid and $r_{c}$ is the radius of the cell. We can also assume that the particles travel with almost no acceleration. As the particle travels, its acceleration quickly decreases and approaches zero. Therefore, the equation above simplifies as follows:

(3) $$\frac{r\omega^{2}\Delta \rho }{\rho_{c}}-\frac{6\pi \mu r_{c}u_{ct}}{m}-2\omega u_{ct}=0$$

The cells therefore reach a terminal velocity, $u_{ct}$, that can be described as:

(4) $$u_{ct}=\frac{r\omega^{2}\Delta \rho m}{\rho_{c}6\pi \mu r_{c}-2\omega m}=\frac{r\omega^{2}\Delta \rho \;V_{c}}{6\pi \mu r_{c}-2\omega m}=\frac{2}{9}\frac{r_{c}{}^{2}\omega^{2}\Delta \rho \;}{\mu }\;r-\frac{\omega \Delta \rho \rho_{c}}{2}r$$

We then integrate *u_ct_* = *dR*/*dt* to estimate the time-dependent radial location *R*(*t*) of cell from its initial location R_0_ to the trap location on the circumference of the device.

(5) $$R\left(t\right)=R_{0}exp\left[\left(\frac{2}{9}\frac{r_{c}{}^{2}\omega^{2}\Delta \rho \;}{\mu }-\frac{\omega \Delta \rho \rho_{c}}{2}\right)t\right]$$

**Figure 5:**
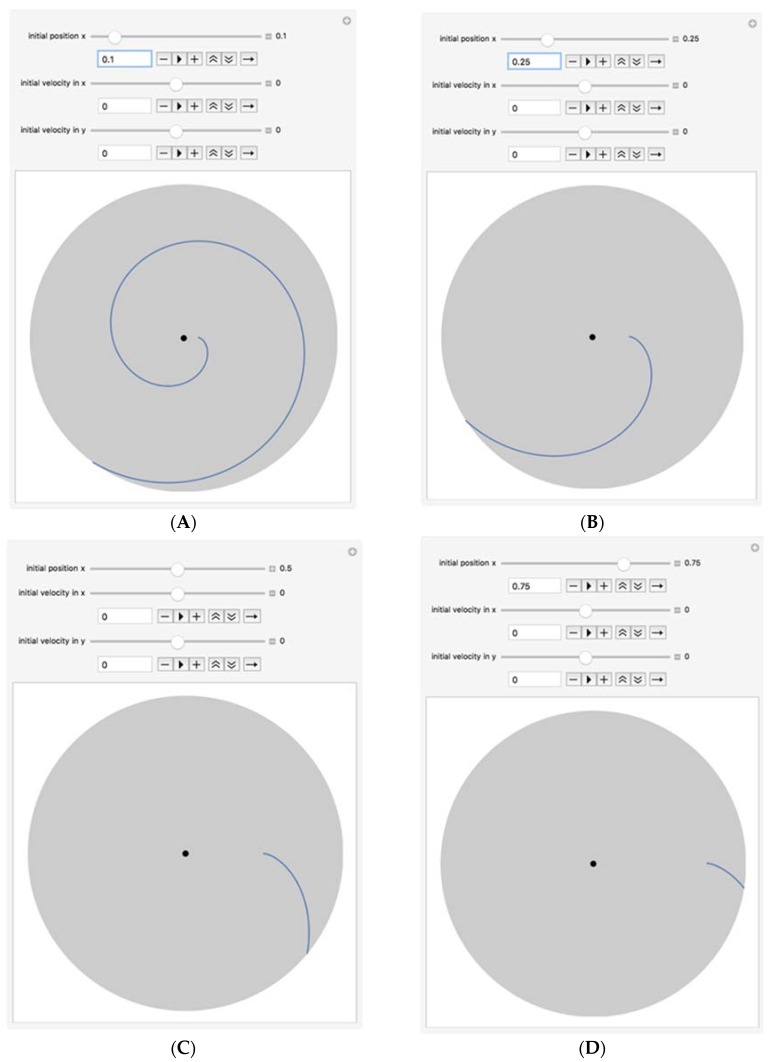
Cell/bead path lines for various initial location of cells. (**A**) From Initial Position Ro = 0.4 cm; (**B**) From Initial Position Ro = 1 cm; (**C**) From Initial Position Ro = 2 cm; (**D**) From Initial Position Ro = 3 cm.

Figure 5 shows cell motion profiles with various starting locations. The trap time for cells from its initial location R_0_ to the trap location R_trap_ is evaluated by $t_{trap}={\left(\frac{2}{9}\frac{r_{c}{}^{2}\omega^{2}\Delta \rho \;}{\mu }-\frac{\omega \Delta \rho \rho_{c}}{2}\right)}^{-1}lnR_{trap}/R_{0}$. For a fluid containing 5000 cells, each occupying a space of 50 μm along the circumference of the disk, N = 5000 and $r_{c}$ = 50. If the fluid is water (or can be approximated as water), $\mu \approx 0.001\;Pa.s,\;R_{cell}$ for breast tumor cells like MCF-7 is ≈0.00001 m, $\rho_{cell}\approx 1050$ kg/m^3^, the following combinations of angular velocities and times to reach the outer radius can be computed (see in Table 1):

Once the cells reach the traps, they are expected to settle on the sides of the cell trap (as predicted by modeling). The fluid, on the other hand, can travel through the drainage channel. Liquid will flow through the channel as long as the pressure due to the centrifugal force is larger than the capillary pressure:

(6) $$\Delta P_{cap}=\frac{2\sigma }{d_{H}}\;cos\theta$$

where σ is the surface tension, $d_{H}$ is the hydraulic diameter of the capillary channel and θ is the equilibrium contact angle that the liquid–air interface forms with the walls of the channel ($\theta \sim 90^{{}^{\circ}}$). The external pressure head, or the pressure due to the centrifugal force, can be described as follows:

(7) $$\frac{dP_{cen}}{dr}=\rho \omega^{2}r$$

Integrating to obtain the pressure difference yields:

(8) $$\Delta P_{cen}=\rho \omega^{2}\left(r_{2}-r_{1}\right)\frac{\left(r_{2}+r_{1}\right)}{2}=\rho \omega^{2}\Delta r\bar{r}$$

where $\bar{r}=\frac{\left(r_{2}+r_{1}\right)}{2},\;\rho$ is the density of the fluid and ω is the angular frequency in rad/s. There exists a critical frequency, $\omega_{c}$, when the liquid will freely flow through the capillary drainage channel, and it can be found when $\Delta P_{cen}\geq \Delta P_{cap}$. This frequency is referred to as the breakthrough frequency or the burst frequency and can be described as follows:

(9) $$\omega_{c}=\sqrt{\frac{2\sigma \;cos\theta }{\rho \;\bar{r}\;\Delta r\;d_{H}}}$$

For the relevant parameters, the minimum value of $\omega_{c}$ was found to be 10 rad/**s** (~100 rpm). Kellogg et al. [13] proposed a different relationship to describe the capillary pressure $P_{cap}$:

(10) $$\Delta P_{cap}=\frac{4\sigma \;sin\theta }{{\left(D_{h}\right)}^{n}}$$

This equation accounts for variations of channel cross sections, which could be useful given the inconsistent nature of the PDMS channel geometry as such high aspect ratios as those used in this study. For a rectangular cross section, n = 1.14. The hydraulic diameter, $D_{h}$, is defined as 4A/P where A is the cross-sectional area of the channel and P is its wetted perimeter. Therefore, the new requirement for flow through the channel is

(11) $$\rho \omega^{2}\Delta r\bar{\;r}>\frac{4\sigma \;sin\theta }{{\left(D_{h}\right)}^{n}}$$

We derive a new expression for the burst frequency:

(12) $$\omega_{c}=\sqrt{\frac{4\sigma \sin\theta }{\rho \;r\;\Delta r\;{\left(D_{h}\right)}^{n}}}$$

**Figure 6:**
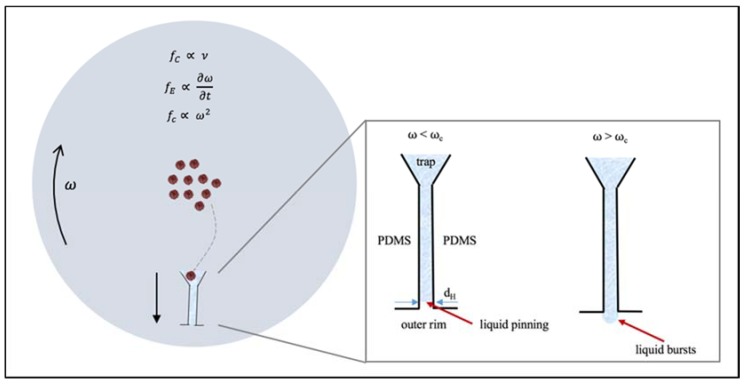
Top view. Forces on the MCD as it spins at an angular velocity, ω. The cells move from the center of the disk towards the traps on the circumference by taking a radial path. Liquid traveling at a speed ν (= dr/dt) down the drainage channel will be exposed to the centrifugal force *f_c_*, the drag force *f_d_* and the Coriolis force, *f_C_*. Inset: When the disk rotates at $\omega <\omega_{c}$, the liquid is “pinned” at the junction since the capillarity pressure is greater than the centrifugal pressure. When the cells have reached their traps, the rotational frequency is increased to $\omega >\omega_{c}$, and the liquid bursts, as the centrifugal pressure exceeds the capillary pressure.

Based on these parameters we calculated the minimum value of $\omega_{c}$ was found to be 15 rad/s (~150 rpm). Figure 6 shows schematic of liquid burst at the burst frequency. Finally, the average fluid velocity in the channel can be obtained by centrifugal theory and can be described as:

(13) $$U=\frac{\rho \;\omega^{2}\;r\Delta r\;{\left(D_{h}\right)}^{2}}{32\;\mu L}$$

where L is the length of liquid in the capillary channel. Accordingly, the volumetric flow-rate (Q) can be described as:

(14) Q=nUA

where n is the number of drainage channels and A is their cross-sectional area. Even though the problem was analyzed from a moving frame of reference, it was important to evaluate the fluid velocity in order to assess the shear rates on the cells. From computational analysis using COMSOL Multiphysics (Burlington, MA, USA), it was found that, for rotational frequencies below 50 rad/s, the maximum shear rates experienced by the cells are 398 s^−1^. This is a positive result, since cells are exposed to shear rates of similar ranges in vivo [14]. This modeling shows that at the calculated burst frequencies (ranging from 10–15 rad/s), the cells should be exposed to shear rates that are well within physiological limits. At these speeds, the cells should take between 5 and 25 min to reach their traps, as determined mathematically.

**Figure 7:**
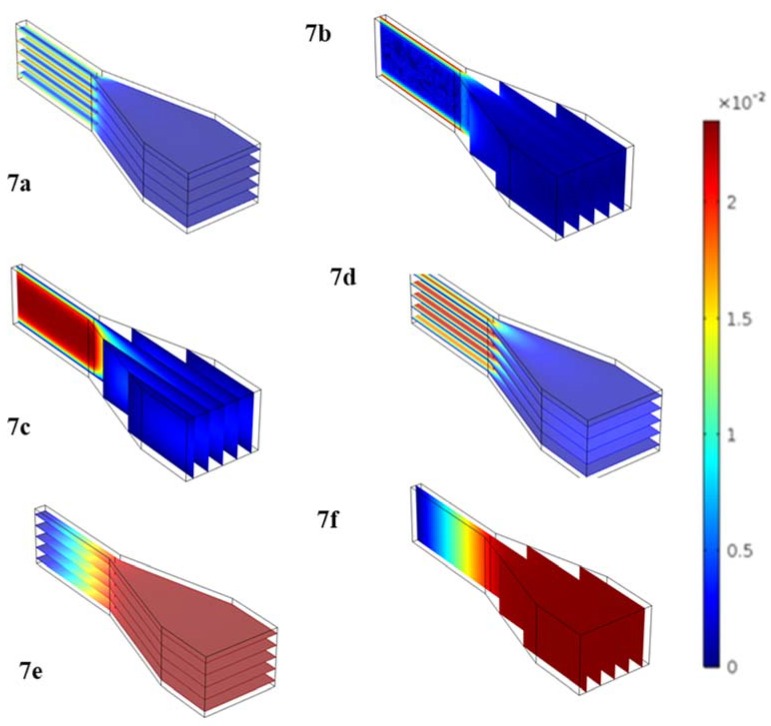
3D Flow simulations generated in COMSOL Free and Porous Media Flow Package to simulate flow through the basic functional unit of the MCD (funnel-shaped trap and connected microchannel). We show shear rates (**a**,**b**), velocity (**c**,**d**) and pressure (**e**,**f**) profiles across one capture well and drainage channel.

Figure 7 shows computationally-obtained models of the shear rates (Figure 7a,b), velocity (Figure 7c,d) and pressure (Figure 7e,f) profiles across one groove and into one capillary channel. To generate these images, the modular geometry was imported from Solidworks (Dassault Systèmes, Vélizy-Villacoublay, France) and 3D flow simulations were carried out in the *Free and Porous Media Flow Package*. These figures show that with the current device geometry and anticipated spin frequency, the shear force the cells would experience on the device would remain below physiological levels. This was critical to ensure cell integrity during operation.

**Figure 8:**
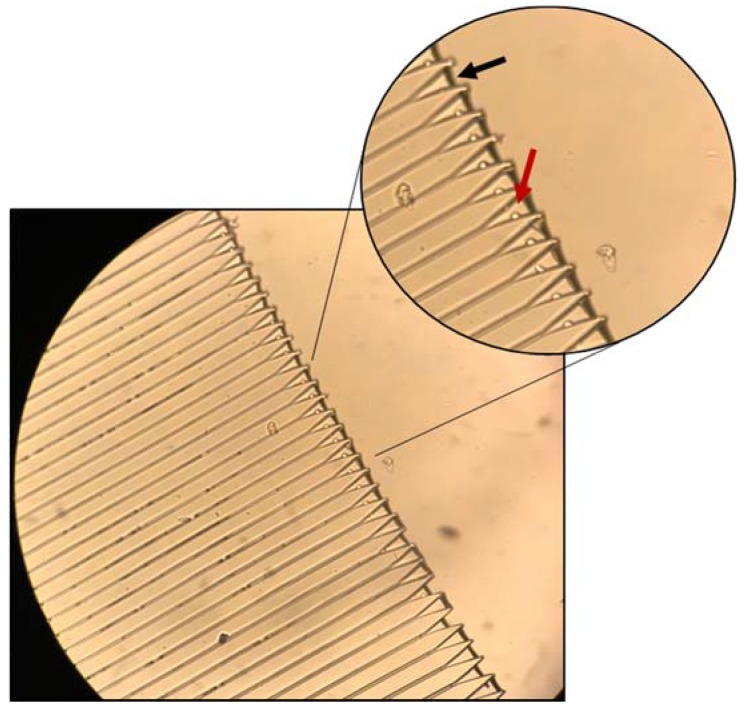
MCD loaded with polystyrene microbeads (15 µm). Maximal capture efficiency was 65% at 150 rpm and 15 min run time. Scale bar: 120 µm; inset scale bar: 80 µm.

**Experimental Investigation of the Centrifugal Device:** Initial testing was performed with spherical polystyrene microparticles designed to mimic the density and size of our target cells. We evaluated 1 mL samples consisting of 2–150 beads/µL at rotational frequencies ranging from 150–300 rpm for time intervals ranging from 5 min to 25 min. Figure 8 shows an example of bead capture. This micrograph was taken with a Leica DM5500B (Leica Microsystems, Buffalo Grove, IL, USA)at 20× magnification after running the sample on the MCD for 15 min at 200 rpm. It is important to notice that, as predicted by the mathematical modeling of the cell’s radial path toward the trap, the particles rest on the side of the funnel-shaped trap (red arrow), rather than clogging the middle of the trap at the channel entrance. This indicates that the path taken by the particles is not solely radial, but curved as previously discussed. Additionally, it can be observed that the liquid level is at the entrance of each trap (black arrow). This is because the centrifugal pressure is not large enough to push the remaining fluid through the drainage channel. Since the liquid is never fully drained, the trap’s content remains submerged even after drainage. This result is important when considering that this chip was designed to host live cells, not beads. The cells require liquid environments and would suffer osmotic imbalances if the fluids were to be completely drained.

**(a) Flow and Capture of Polystyrene Microbeads:** The maximal trap occupancy rate is defined as the occupied traps divided by total traps. This makes the particle capture efficiency, $\eta_{c}$, important where

$$\begin{array}{c} Capture\;Efficiency=\frac{\#\;wells\;occupied\;by\;a\;single\;particle}{\#\;loaded\;particles}\;\% \end{array}$$

$$Trap\;Occupancy\;Rate=\frac{\#wells\;occupied\;by\;a\;single\;particle}{\#\;well\;total}\;\%$$

**Figure 9:**
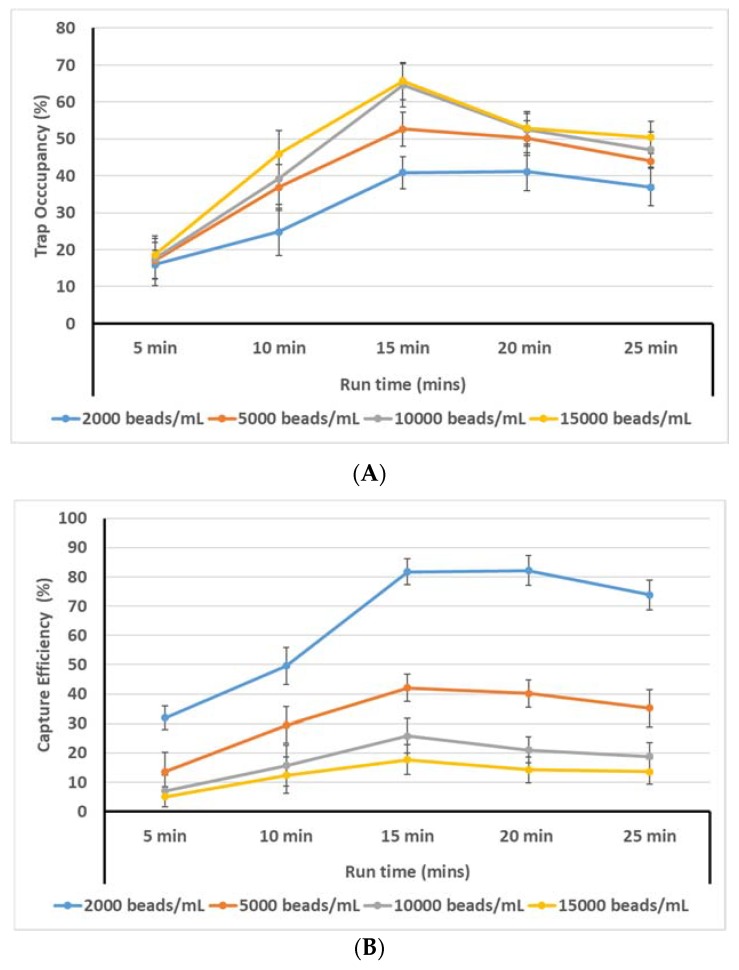
(**A**) Trap occupancy rate vs. run time for different loading concentrations. (**B**) Bead capture efficiency vs. run time for different loading concentrations.

Here, the reported rates include only wells with single particles/cells. The incidence of multiple particles or cells in the wells was very low. We have added a discussion of this in the text. The highest trap occupancy observed was 65% and was observed at 200 rpm with 100 beads/µL and 15 min of run time (Figure 9A). At shorter run times (5 and 10 min), it is likely the particles do not have sufficient time to travel to the traps. With longer run times (20 and 25 min), most particles have already reached their path and the extra run time may cause them to be displaced by the continued motion, decreasing the trap occupancy. We observed that higher loading particle concentration leads to greater trap occupancy. This is an intuitive finding: the greater the number of particles loaded, the greater the trap occupancy. However, this also leads to lower capture efficiency (Figure 9B). The goal of this device is to perform single-cell isolation and subsequent analysis, thus it is important that the greatest possible fraction of loaded particles is trapped.

Figure 9B also shows that a loading concentration of 15,000 beads/mL yields the highest trap occupancy rate (65.65%); however, the capture efficiency for the same combination of loading concentration and run time is $\eta_{c}$(15,000 beads/mL, 15 min) = 17.5%. On the other hand, for the 2000 beads/mL loading concentration at the 15 min run time, the trap occupancy is 40.85%, meaning that 1630 cells were captured. Thus $\eta_{c}$(2000 beads/mL, 15 min) = 1630 / 2000 = 81.7%. These results point to two diverging trends: (i) the higher the initial loading concentration, the greater the trap occupancy, but the lower the capture efficiency; (ii) the lower the initial loading concentration, the lower the trap occupancy but the higher the capture efficiency. Both of these two findings can be desirable depending on the application of the device: if a rare sample is being analyzed and it is important to obtain cell-specific information for every cell in the sample, it may be more useful to use lower loading concentrations so that the number of captured cells is maximized. On the other hand, if the cells are not part of a rare sample, then it may be more desirable to use higher loading concentrations in order to optimize the efficiency of the device and minimize loss of assay reagents. Therefore, rather than recommending a single optimum loading, we believe there are two optimal use cases based on the goals of the user: maximum detection of very rate cells, or maximum throughput. Rare cell detection would rely on maximizing the capture rate, whereas maximum throughput would be achieved via maximizing trap occupancy.

**(b) Flow and Capture of MCF-7 Cells:** For the purposes of this investigation, the optimal combination of run time and loading concentration was chosen based on the graph in Figure 9A,B in order to strike a compromise between maximizing the device’s efficiency and the cell capture rate. Thus, MCF-7 cells were loaded at concentrations of 5000 cells/mL and were exposed to centrifugal motion for 15 min. Ensuring 100% trap occupancy would require a much higher number of beads to be loaded and would also lead to an excess number of beads collecting around the perimeter of the device.

We characterized the device using 1 mL samples of 5 cells/µL. Cells present additional challenges compared to the beads used during the first stage of experimental optimization: adherence and deformability. To preserve the integrity of the cells, the MCD was optimized to handle cells with flow speeds and shear conditions below those experienced in the blood circulation to promote cell health. The peak flow speed of 2 cm/s, as determined through computational modeling occurs at the entrance of the trap and is much lower than the speeds experienced by cells in the circulation.

**Figure 10:**
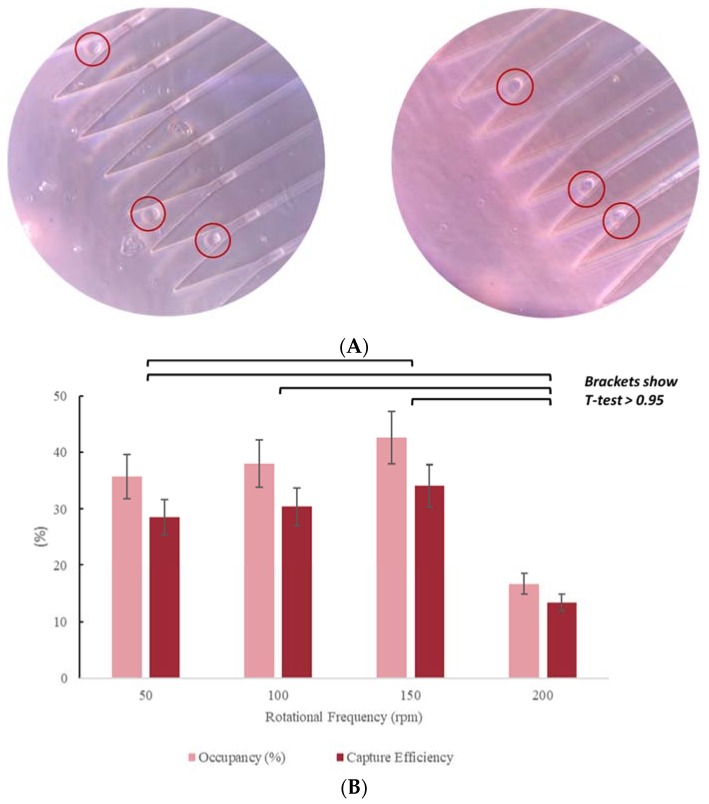
(**A**) MCD loaded with MCF-7 cells. Micrographs obtained with Leica DM5500B, at 20X. Trap occupancy rate: 42%; initial loading concentration: 5000 cells/ mL. Scale: 30 µm is the distance from one capture well to other. (**B**) Occupancy rate and capture efficiency as functions of rotational speed.

The efficiency increased with increasing rotational frequency until a frequency of 150 rpm is reached. Beyond 150 rpm increased frequencies caused decreased capture rates (Figure 10). Reduced capture at high rotational frequencies is likely a result of more frequent cell lysis under higher shear stress. This hypothesis was confirmed by the presence of uncharacterized debris observed in the MCD following assays run at $\omega_{o}>150\;\mathrm{rpm}$. Therefore, 150 rpm was selected as the optimal rotational frequency to provide high capture efficiency. At 150 rpm for 15 min, the trap occupancy was found to be 42%, with a corresponding capture efficiency of 34%.

Unlike in the bead experiments where beads were captured along the side of the capture well, cells were observed closer to the mouth of the drainage channel. We hypothesize this is because the cells are more deformable than the polystyrene beads. This does not interfere with operation of the MCD. Due to the lower occupancy rate, approximately 40% of the 4000 drainage channels are always empty and unclogged, allowing for successful drainage. We also observed that most cells did not deform into the channels but rather were individually trapped in the device or adhered to the loading area of the device.

**Figure 11:**
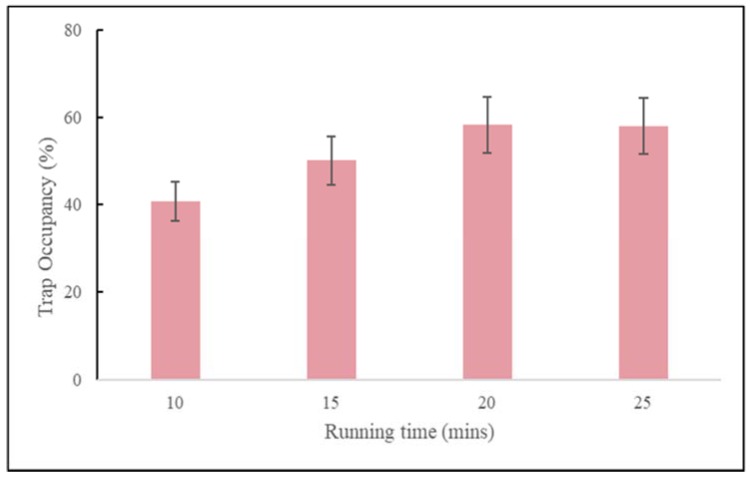
Occupancy rate as functions of running time (mins) for MCF-7 cells loaded at a concentration of 5000 cells/mL, at an operating rotational frequency of 150 rpm.

As observed with the polystyrene beads, there is a trade-off between capture efficiency and trap occupancy. Loading a higher number of cells leads to greater trap occupancy but lower capture efficiency. Both occupancy rate and capture efficiency were optimized together with respect to both rotational frequency and spin time. We tested 10, 15, 20, and 25-min spin times and found that whereas the polystyrene beads worked best with a 15-min spin time, the cells worked best with a 20-min run time (Figure 11). We also tested cell viability by performing time course experiments looking for cell loss after capture. We observed close 100% viability by microscope cell count observations.

One hypothesis for the increased running time is that the properties of the cells affect their transport, such as proteins on the cell surface which influence adhesion. Additionally, literature has described the ability for circulating cells to develop adherence towards the walls of vessels under certain flow conditions, as found through studies aiming to reproduce circulatory flow [15]. The drop off in both occupancy rate and capture efficiency from 150 rpm to 200 rpm could be attributed to the increase in shear forces acting on the cell, potentially causing cell lysis. This theory is supported by the observation of, not only fewer cells in the traps, but also fewer cells stuck in the loading area of the device.

It is important to discuss the observed capture efficiency in our device in reference to the performance of other devices. Maria Antfolk et al.’s work [6] showed the capture efficiency to be 75%–100% for cancer cells, however, the device only processed 16 cells/min–2 cells/min for 5700 traps. Hence, their device had to run for 48 h for 100% capture efficiency and 8 h for 75% capture efficiency. Similarly, other studies [16,17] also showed higher capture efficiencies, however, process times were more than 20 h. Mach et al. [18] showed capture efficiency of 20% when 500 cells were captured, not at a single cell level, in just over 2 min. Our device sorted 5000 cells for 4000 traps in just 15 min with 35% capture efficiency where, unlike previous studies, we did not discount any the smaller or deformed cells. In our experiments, a population of MCF-7 cells deformed and passed through the 10 µm × 100 µm rectangular traps. The central idea of our approach is to offer a centrifugal device which is suitable to rapidly process large number of cells once it is optimized for each cell line or application. It is the intent of this manuscript to highlight the importance of various parameters which can be used for such an optimization.

**Capture of Artificial CTC Clusters:** Due to the increasing clinical and research interest in the role of CTC clusters in cancer metastasis, we fabricated a second version of the device with 200 µm width traps connected to 30 µm wide channels. We used a limited-trypsinization technique of MCF-7 cell culture to make artificial CTC clusters from clusters of MCF-7 cells. CTC clusters are commonly considered to consist of 2 to 30 cells of 15–25 µm in diameter [19]. The intent of this design was to encourage the flow of individual cancer cells and off-target cells through the drainage channel while retaining only cell clusters in the well-trap. We recognize that it is possible to add an outer concentric ring of the trap dimensions previously described to capture the cancer cells after they flow through the CTC cluster trap wells. The design of the CTC cluster capture wells was also designed to occlude flow through the channel after a cluster had been captured, thereby creating a positive pressure around the well to prevent individual cells from being trapped in the well.

**Figure 12:**
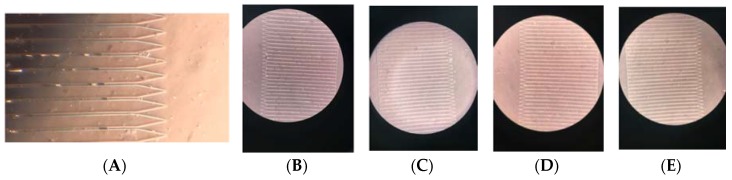
CTC Cluster Images: The cluster occupancies (**A**) 15%, (**B)** 21%, (**C**) 8%, (**D**) 8%, (**E**) 27%. Scale: 30 µm is the distance from one capture well to another.

With this modified geometry, the MCD can process 2 mL of blood in 10 min (12 mL/h). The new geometry comprises of a PDMS disk of diameter 8 cm with 1000 traps housed along its circumference. We observed a range of 7%–27% trap occupancy using the cell clusters (Figure 12). The trapped clusters themselves ranged from 2–9 cells. We believe there may be a reduction in trap occupancy compared to the occupancy achieved with individual cells due to shear-induced fragmentation of the cell clusters. The structural integrity of our model clusters vs. true CTC clusters is unknown. We do observe many individual cells in the drainage channel of the traps, some of which may be due to fragmented cell clusters.

## 4. Conclusions

Here we presented a rapid technique for the massively parallel separation of thousands of cells. We believe there is much more scope to exploit this device’s design. Its transparent construction makes on-device imaging simple. Further, as demonstrated by the MCF-7 vs. MCF-7 cluster capture, variable capture well geometries allow for the differential capture of different target cells based on size. The ability to sort cells based on surface markers (via imaging studies) in addition to size and deformability makes this device ideal for analysis of heterogeneous cell populations. The cluster images show how individual MCF-7 cells can easily pass through the drainage channels designed for cell clusters without rupture. It should also be possible to integrate multiple geometries on a single ring-device to capture more than one cell type, or, multiple rings of capture wells inside each other with decreasing capture well size as a means of assessing deformability or size. The rapid size-based filtration achieved by a series of concentric rings with decreasing trap size may be particularly useful for the separation of cancer cell clusters and circulating tumor cells (CTCs) from whole blood, as these species are much larger than red blood cells and platelets. There may be sufficient difference in size and deformability to also resolve leukocytes from CTCs. One final application may be based on using the spinning and draining as a washing system to add a series of reagents to many single-cell reactions in parallel, such as a surface protein ELISA (enzyme-linked immunosorbent assay).

## Figures and Tables

**Table 1:** Time for the cells to align along the outer diameter computed with respect to angular velocity.

Time, t_outer_ (s)	Angular Velocity, Ω (rad/s)	Velocity (rpm)
22758.43	1.047	10
5689.61	10.47	100
1423.76 (24 min)	20.93	200
423.8 (6.4 min)	31.42	300
56.90	104.7	1000
14.22	209.4	2000
